# A protocol for urine collection and storage prior to DNA methylation analysis

**DOI:** 10.1371/journal.pone.0200906

**Published:** 2018-08-24

**Authors:** J. Bosschieter, S. Bach, I. V. Bijnsdorp, L. I. Segerink, W. F. Rurup, A. P. van Splunter, I. Bahce, P. W. Novianti, G. Kazemier, R. J. A. van Moorselaar, R. D. M. Steenbergen, J. A. Nieuwenhuijzen

**Affiliations:** 1 Cancer Center Amsterdam, Department of Urology, VU University Medical Centre, Amsterdam, the Netherlands; 2 Cancer Center Amsterdam, Department of Surgery, VU University Medical Centre, Amsterdam, the Netherlands; 3 BIOS Lab on a Chip group, University of Twente, Enschede, the Netherlands; 4 Cancer Center Amsterdam, Department of Pathology and Molecular Biology, VU University Medical Centre, Amsterdam, the Netherlands; 5 Cancer Center Amsterdam, Department of Pulmonology, VU University Medical Centre, Amsterdam, the Netherlands; 6 Cancer Center Amsterdam, Department of Epidemiology and Biostatistics, VU University Medical Centre, Amsterdam, the Netherlands; Institut de genomique, FRANCE

## Abstract

**Background:**

Urine poses an attractive non-invasive means for obtaining liquid biopsies for oncological diagnostics. Especially molecular analysis on urinary DNA is a rapid growing field. However, optimal and practical storage conditions that result in preservation of urinary DNA, and in particular hypermethylated DNA (hmDNA), are yet to be determined.

**Aim:**

To determine the most optimal and practical conditions for urine storage that result in adequate preservation of DNA for hmDNA analysis.

**Methods:**

DNA yield for use in methylation analysis was determined by quantitative methylation specific PCR (qMSP) targeting the *ACTB* and *RASSF1A* genes on bisulfite modified DNA. First, DNA yield (*ACTB* qMSP) was determined in a pilot study on urine samples of healthy volunteers using two preservatives (Ethylenediaminetetraacetic acid (EDTA) and Urine Conditioning Buffer, Zymo Research) at four different temperatures (room temperature (RT), 4°C, -20°C, -80°C) for four time periods (1, 2, 7, 28 days). Next, hmDNA levels (*RASSF1A* qMSP) in stored urine samples of patients suffering from bladder cancer (n = 10) or non-small cell lung cancer (NSCLC; n = 10) were measured at day 0 and 7 upon storage with and without the addition of 40mM EDTA and/or 20 μl/ml Penicillin Streptomycin (PenStrep) at RT and 4°C.

**Results:**

In the pilot study, DNA for methylation analysis was only maintained at RT upon addition of preserving agents. In urine stored at 4°C for a period of 7 days or more, the addition of either preserving agent yielded a slightly better preservation of DNA. When urine was stored at -20 °C or -80 °C for up to 28 days, DNA was retained irrespective of the addition of preserving agents. In bladder cancer and NSCLC samples stored at RT loss of DNA was significantly less if EDTA was added compared to no preserving agents (p<0.001). Addition of PenStrep did not affect DNA preservation (*p*>0.99). Upon storage at 4°C, no difference in DNA preservation was found after the addition of preserving agents (*p* = 0.18). The preservation of methylated DNA (*RASSF1A*) was strongly correlated to that of unmethylated DNA (*ACTB*) in most cases, except when PCR values became inaccurate.

**Conclusions:**

Addition of EDTA offers an inexpensive preserving agent for urine storage at RT up to seven days allowing for reliable hmDNA analysis. To avoid bacterial overgrowth PenStrep can be added without negatively affecting DNA preservation.

## Introduction

Molecular biomarkers are extensively investigated and may contribute to early detection, monitoring and prediction of therapy response in cancer patients [1, 2]. These biomarkers represent genetic and epigenetic events associated with cancer development. One of these events includes DNA methylation. DNA methylation is an epigenetic process involving the addition of a methyl group to cytosine, which mainly occurs in CpG dinucleotides. Hypermethylation in CpG-rich promoter regions can result in silencing of tumour suppressor genes, thereby driving carcinogenesis [1]. Thus, detection of hypermethylated DNA (hmDNA) in bodily fluids such as urine and blood are of interest as an oncological biomarker [3]. Urine has clear advantages over blood as the collection of urine is non-invasive and it is available in large amounts. Moreover, there is no need for qualified personnel to obtain the sample which allows for extramural collection. In bladder cancer patients, the detection of urinary hmDNA has been found to correlate strongly to the presence of bladder cancer [4]. Recently, Reckamp *et*. *al* showed that tumour-derived DNA can also be detected in urine of lung cancer patients [5]. Yet, despite promising results, utilization of urinary hmDNA in clinical practice, and especially in case of extramural collection, is limited by the challenges of preserving urinary hmDNA. Ideally DNA analysis should be performed immediately upon urine collection, since urine contains DNA hydrolysing enzymes (DNases) which greatly accelerate DNA degradation. Yet, immediate analysis is not always possible because molecular analysis requires a specialized laboratory and specialized personnel. Therefore, urine needs to be stored and transported in such a way that DNA preservation is ensured to allow for downstream analysis.

For the purpose of DNA methylation analysis, preservation of DNA is even more important as the need for bisulfite conversion of DNA results in significant degradation of DNA [6]. Bisulfite converts unmethylated cytosines into uracil, whereas methylated cytosines remain unaltered, such that differentiation between methylated and unmethylated DNA is possible using PCR-based methods.

DNA quality in urine samples is mainly influenced by the time interval from sampling to analysis and storage temperature. The use of preserving agents may retain urinary DNA for a longer period of time [7]. Ethylenediaminetetraacetic acid (EDTA) is a well-established chelating agent that binds ions required for DNase activity [8]. Consequently, adding EDTA might reduce DNA degradation. Furthermore, the process of bacterial uptake and recombination in case of bacterial contamination can reduce DNA yield [9, 10]. By the addition of antibiotics, this process can be prevented.

In this 2-step study, we analyzed the preservation of urinary DNA for methylation analysis in healthy volunteers and in patients using urine stored at different temperatures and for different time periods with and without the addition of preserving agents.

## Materials and methods

This pilot and patients sample study was approved by the Medical Ethics Review Committee of the VU University Medical Centre and informed written consent was obtained from all participants.

### Sample collection and storage

#### Pilot study

**Fig 1:**
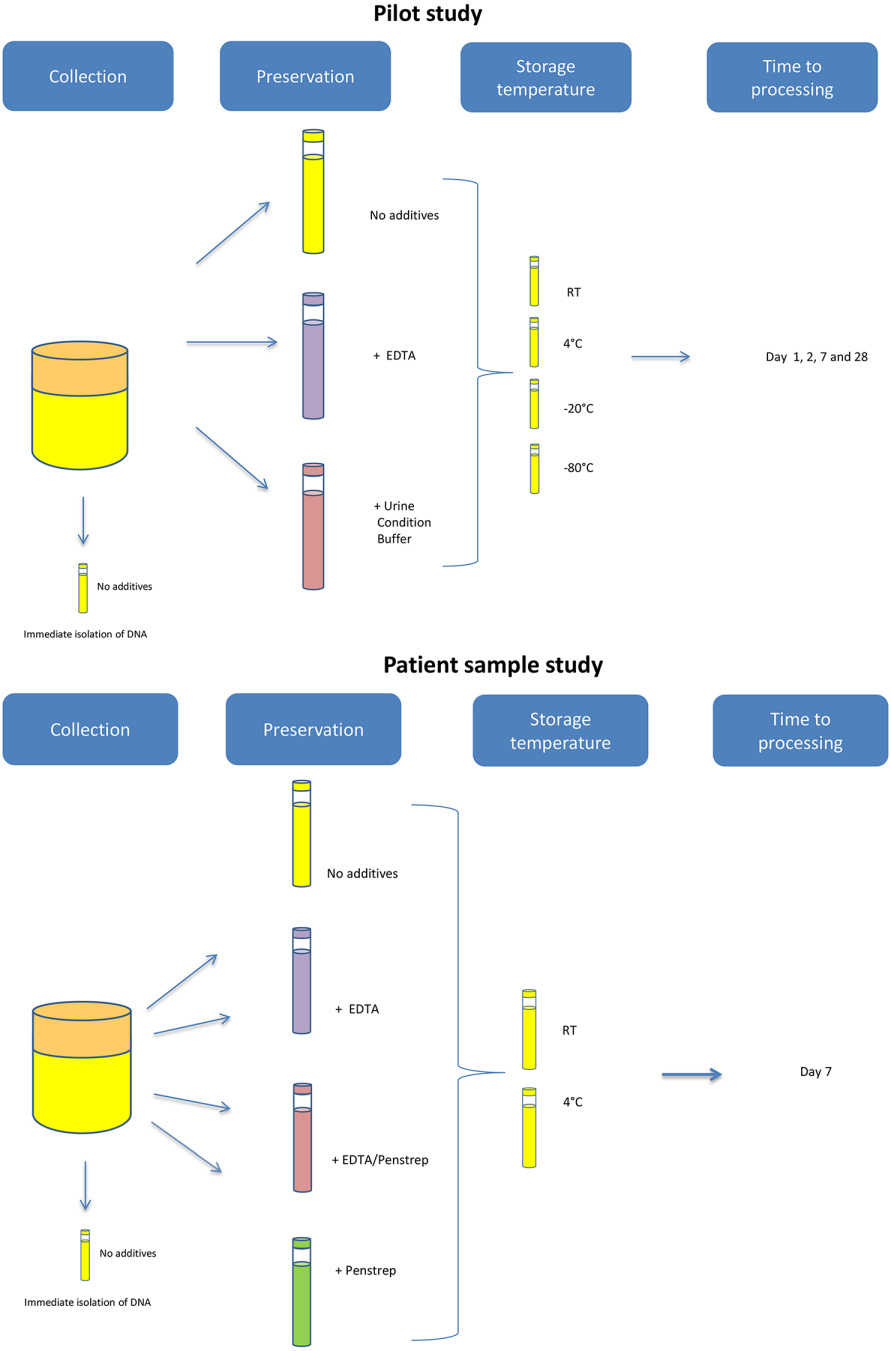
Schematic overview of sample handling in volunteers (*n* = 3) and patients (non-small cell lung cancer *n* = 10, bladder cancer, *n* = 10).

To preselect storage conditions for further testing on patient materials, the DNA yield for methylation analysis was determined in urine collected from three healthy volunteers stored under various conditions. All three volunteers provided written, informed consent to study participation. Each urine sample was divided into four equal volume aliquots of which one aliquot was used for immediate DNA isolation. DNA was extracted from native urine. Preserving agents, EDTA (final concentration of 40mM) and 70 μl/ml Urine Conditioning Buffer^™^ (Zymo Research, Orange, CA, U.S.A.), were added to aliquot two and three, respectively. No preserving agent was added to aliquot four. Thereafter samples were stored at different temperatures (room temperature (RT), 4°C, -20°C and -80°C) and processed on days 1,2, 7 and 28 (Fig 1).

#### Patient sample study

Urine samples were provided by bladder cancer and non-small cell lung cancer (NSCLC) patients at the VU University Medical Center Amsterdam and the Amstelland Hospital Amstelveen between November 2016 and May 2017. Samples of bladder cancer patients (*n* = 10) were collected prior to transurethral resection and samples from NSCLC patients prior to lobectomy (*n* = 10). All specimens were divided into 9 equal volume aliquots. Preserving agents included EDTA in a final concentration of 40mM and/or 20ul/ml penicillin-streptomycin (PenStrep). Storage temperatures were RT or 4°C. Processing was done immediately after collection (day 0) and at day 7 (Fig 1).

### DNA isolation

DNA was isolated using the Quick-DNA^™^ Urine Kit (Zymo Research, Orange, CA, U.S.A.) according to the manufacturer’s protocol. In the pilot study aliquots of 10 ml were used, whereas patient’s samples contained equal aliquots of 4-10ml, depending on the original volume. DNA was eluted in 50 μl of elution buffer and stored at -20°C. To determine the preservative effects of EDTA addition on non-bisulfite converted DNA, concentrations of a subset of DNA isolates were measured using the Qubit^™^ dsDNA HS Assay (Invitrogen).

### Methylation specific PCR

To allow for Quantitative Methylation Specific PCR (qMSP) analysis, 40 μl of isolated DNA was treated with bisulfite using the EZ DNA Methylation^™^ kit (Zymo Research, Orange, CA, U.S.A.). DNA isolated from the bladder cancer cell line RT-112, kindly provided by prof. G.J. Peters (VU University Medical Center, Amsterdam, the Netherlands), was used for the generation of a standard curve. Quantitative MSPs of the housekeeping gene β-actin (*ACTB*) and *RASSF1A* were performed as described previously [11, 12].

In short, amplification reactions contained a total volume of 12 μl including EpiTect MethyLight Master Mix (Qiagen), 200 nM of each primer and fluorescent dye-labeled probe, and 2.5 μl bisulfite treated DNA. The amplification reactions were carried out at 95°C for 5 minutes, followed by 45 cycles at 95°C for 15 seconds and 60°C for 1 minute in 96-well plates in an ABI 7500 Fast Real-Time PCR System (Life Technologies, Thermofisher Scientific). Samples with *ACTB* cycle threshold (CT) of >32 (a commonly used threshold for defining unreliable hmDNA analysis) [13] at day 0 were excluded.

### Data analysis

For the pilot study a limited sample size (n = 3) was used and no statistical comparisons were planned.

In the patient sample study, decay in DNA for the purpose of hmDNA analysis was calculated by analyzing the log fold change of the *ACTB* (log2FC_*ACTB*_). The following formula was used:

$${{log2FC}_{ACTB}\;=\;{\text{log}}_{2}\;(2}^{\left[{CT}_{ACTB}(t)-{CT}_{ACTB}(0)\right]})$$ (1)

With CT_*ACTB*_(t) and CT_*ACTB*_(0) the CT values of *ACTB* at time t = 7 days and day 0 respectively.

To define the correlation between *ACTB*-CT and *RASSF1A*-CT at various conditions the following formula was used:

$$\Delta {CT}_{gene,condition\;X}\;=\;{CT}_{gene,condition\;X}(t)-\;{CT}_{gene,condition\;X}(0)$$ (2)

With CT_gene,condition X_ (t) the CT of the gene of interest at time t with condition X.

Prior to combining the samples of NSCLC and bladder cancer patients, differences in the ΔCT_*ACTB*_ and ΔCT_*RASSF1A*_ between both groups were analyzed using the Mann Whitney *U* test. When the differences were not statistically significant, samples of both groups were combined to increase statistical power. The difference between ΔCT_*ACTB*_ and ΔCT_*RASSF1A*_ at various conditions was assessed using Wilcoxon signed-rank tests. Differences between storage conditions were examined using a Friedman test for multiple extractions. When overall significance was observed, post-hoc Bonferroni-corrected analysis was performed using the related-samples Wilcoxon signed-rank test for two-by-two comparisons. The omnibus test for the multiple storage conditions was performed with R statistical software (version 3.3.1) using FSA package [14]. Remaining analyses were performed with SPSS software (SPSS 22.0, IBM, Armonk, NY, USA). All tests were two-sided and a significance level of 0.05 was applied.

## Results

### Pilot study

**Fig 2:**
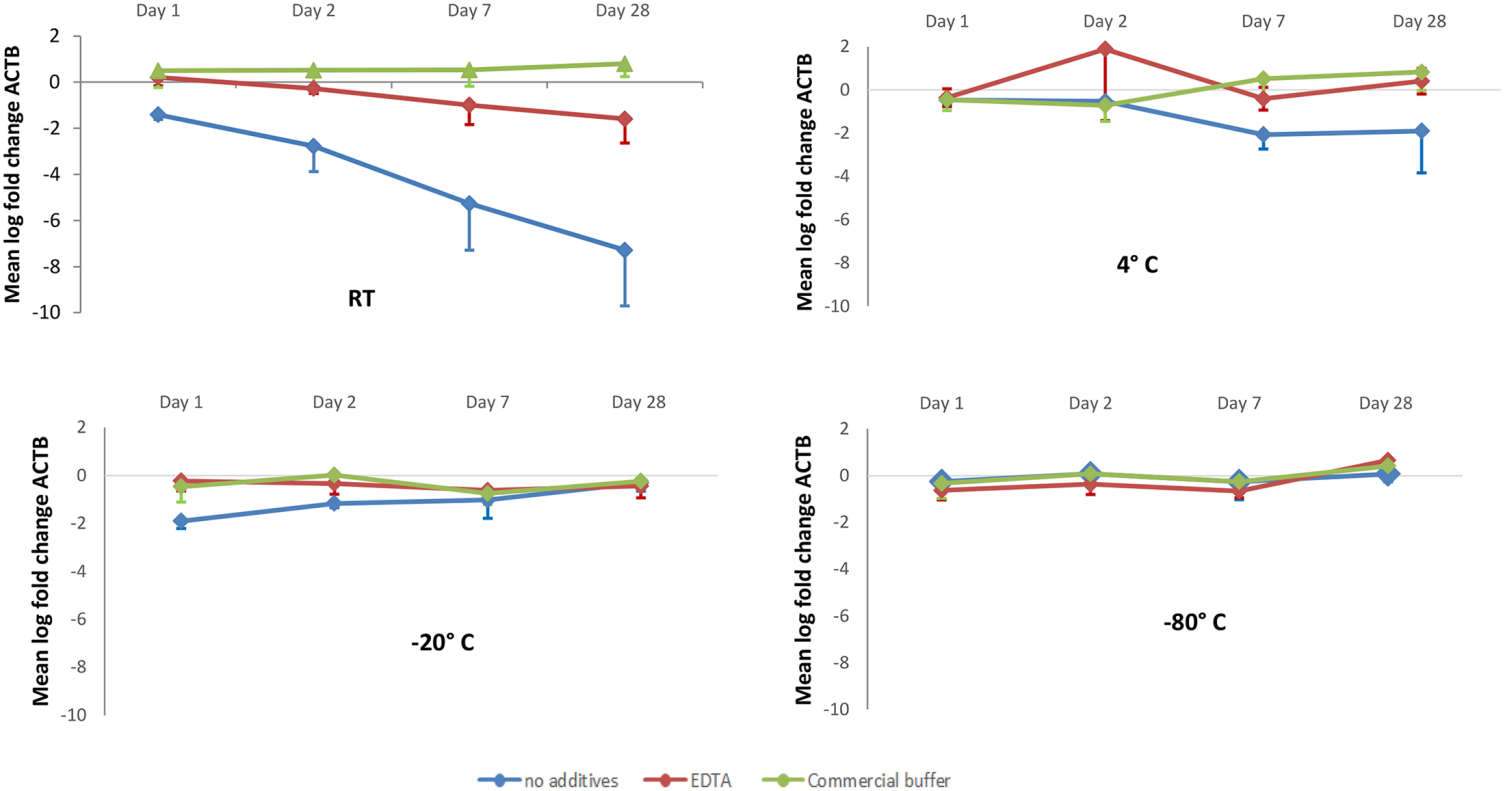
Difference in bisulfite modified DNA measured by mean log fold change of *ACTB* compared to t = 0 after storing urine at room temperature, 4°C, -20°C and -80°C with and without preserving agents. Error bars represent the standard deviation between three urine samples derived from different donors. RT = room temperature; *ACTB* = β-actin; EDTA = Ethylenediaminetetraacetic acid.

Analysis of three aliquoted urine samples stored at RT showed that the addition of both Urine Conditioning Buffer^™^ (Zymo Research, Orange, CA, U.S.A.) and EDTA resulted in better preservation of DNA as compared to no preserving agents (Fig 2, S1 File). None of the samples to which EDTA or Urine Conditioning Buffer^™^ was added had CT_*ACTB*_ > 32. Without preserving agents, one out of three samples exceeded this value at day 2 and another one at day 7. At day 28 all three untreated samples had a CT_*ACTB*_ > 32.

Upon storage at 4°C, DNA was maintained for the first two days, irrespective of the use of preserving agents (Fig 2). After 7 days DNA was better retained if EDTA or the commercial buffer were added, as compared to no preserving agents (Fig 2). At -20°C and at -80°C, degradation of DNA was not observed, regardless of the use of preserving agents (Fig 2).

These results suggest that the addition of EDTA is similarly effective to the commercial buffer in terms of reducing DNA degradation when samples are stored at 4°C. At RT the effect of the commercial buffer and EDTA were comparable for a time period of 7 days. However, after 28 days the commercial buffer seemed superior to EDTA. Nevertheless, we chose to use EDTA for further testing for economic reasons. Furthermore, there was still a comparable efficacy at day 7, which is sufficient to allow for further processing. Storage at room temperature and 4°C are the most practical in routine settings. For these reasons we chose to determine the efficacy of EDTA at RT and 4°C in our further studies in which we used patient samples. In addition, with the possibility of bacterial contamination in mind, the addition of antibiotics (penicillin/streptomycin; PenStrep) was also tested.

### Patient sample study

#### DNA

CT values per sample are given in the S2 File. One patient was excluded from analysis, due to a high CT value (>32) for *ACTB* at day 0. The differences in ΔCT*ACTB* and ΔCT*RASSF1A* between NSCLC samples and bladder cancer samples did not significantly differ at any of the conditions (S1 Table). As of such, samples of both patient groups were combined in further analyses to increase statistical power.

**Fig 3:**
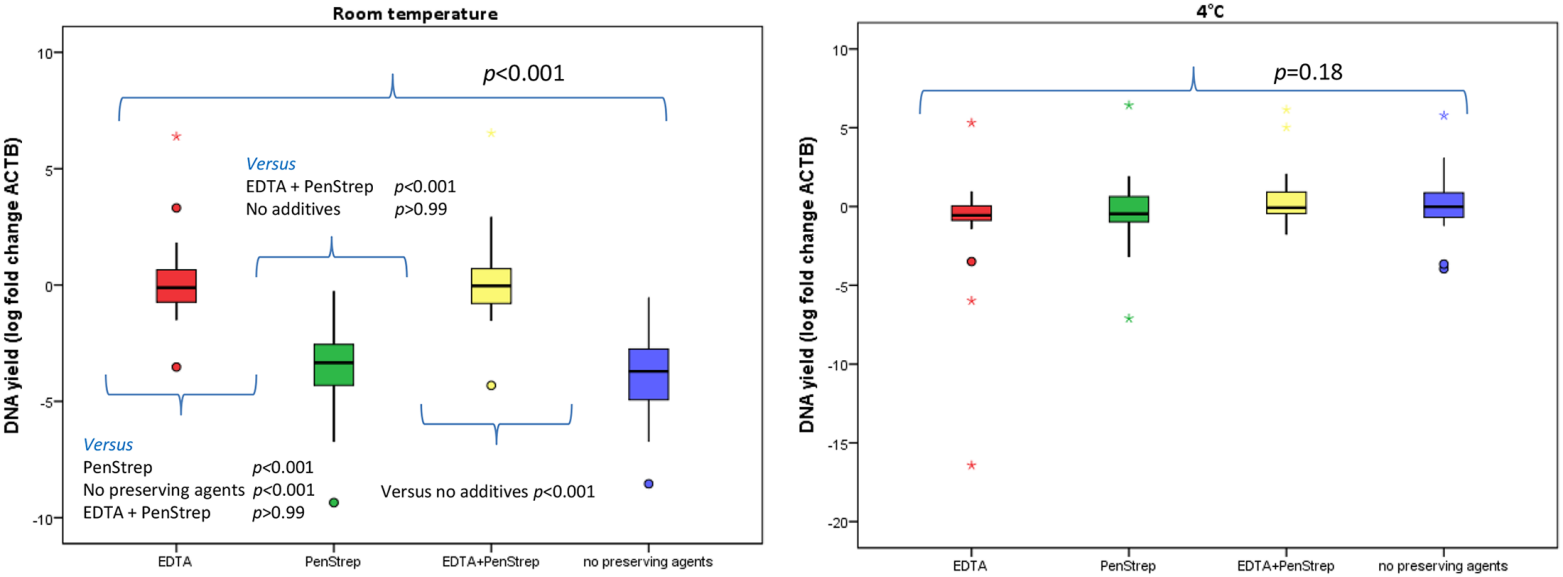
DNA derived from urine samples stored at room temperature and 4°C, after 7 days with and without preserving agents. DNA degradation was measured by the log fold change of *ACTB* at various conditions compared to day 0. Results of the post-hoc analysis of DNA degradation at room temperature are presented above and under the small bars. EDTA = Ethylenediaminetetraacetic acid; PenStrep = Penicillin Streptomycin.

The calculated mean log fold change in *ACTB* levels (*n* = 19) at various conditions are presented in Fig 3. A statistically significant difference was present in the mean log fold change of *ACTB* level between the various conditions when urine samples were stored at RT (Fig 3, *p*<0.001), but not when stored at 4°C (Fig 3, *p* = 0.18).

A post-hoc analysis on differences in DNA degradation for urine stored at RT demonstrated that DNA was significantly better preserved when EDTA was added (Fig 3). Addition of PenStrep did not preserve DNA (*p*>0.99). A similar preservative effect of EDTA was found for non-bisulfite modified DNA (S2 Table).

A total of seven samples had an CT_*ACTB*_ > 32. Conditions of these samples included: PenStrep at RT (*n* = 3), no preserving agents at RT (*n* = 3) and PenStrep at 4°C (*n* = 1). None of the samples to which EDTA was added were associated with a CT_*ACTB*_ >32.

#### hmDNA

**Fig 4:**
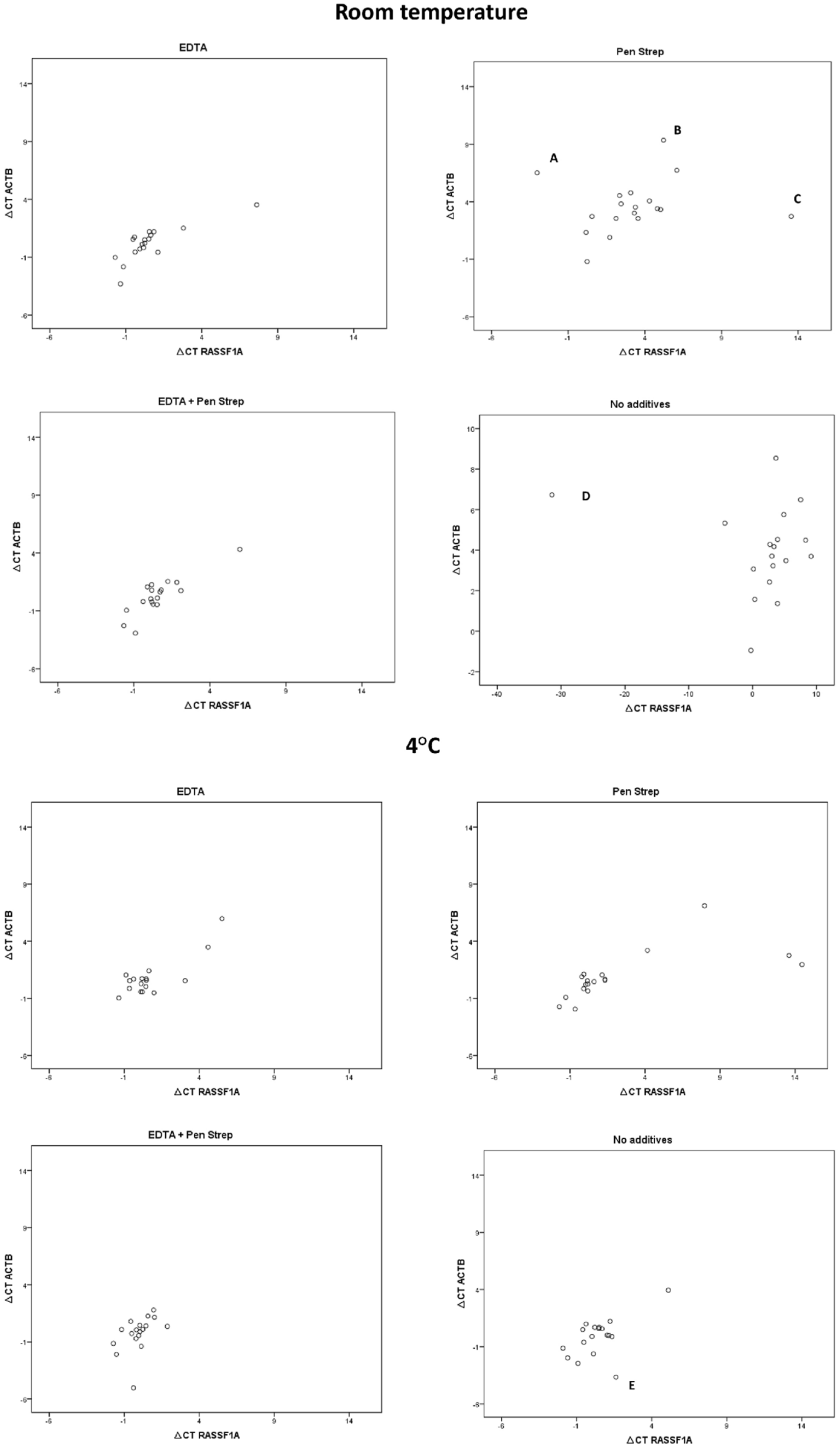
Scatter plots showing ΔCT of β actin (*ACTB*) and the ΔCT of methylated *RASSF1A* with or without various preserving agents (preservatives) at room temperature and at 4°C after 7 days of storage. The outliers are marked with A-E.

The difference between ΔCT_*ACTB*_ and ΔCT_*RASSF1A*_ at various conditions is graphically presented in scatter plots (Fig 4).

There was no statistically significant difference in the ΔCT_*ACTB*_ and ΔCT_*RASSF1A*_ in any of the conditions (Table 1).

**Table 1:** The difference between the ΔCT_*ACTB*_ and ΔCT_*RASSF1A*_ (day 7) for various conditions.

**Condition at RT**	**ΔCT_*RASSF1A*_** _**median (IQR)**_	**ΔCT_*ACTB*_** _**median (IQR)**_	***p*-value** **(Wilcoxon signed-rank test)**
**EDTA**	0.16 (-0.53–0.64)	0.22 (-0.58–0.90)	0.7
**PenStrep**	3.07 (0.55–4.81)	3.33 (2.55–4.54)	0.5
**EDTA + PenStrep**	0.19 (-0.39–0.80)	0.11 (-0.46–1.07)	0.2
**No preserving agents**	3.19 (0.13–4.88)	3.71 (2.42–5.33)	0.4
**Condition at 4°C**	ΔCT_*RASSF1A*_ _median (IQR)_	ΔCT_*ACTB*_ _median (IQR)_	*p*-value (Wilcoxon signed-rank test)
**EDTA**	0.21 (-0.63–0.99)	0.55 (-0.42–1.05)	0.4
**PenStrep**	0.19 (-0.21–1.33)	0.53 (-0.35–1.12)	0.16
**EDTA + PenStrep**	-0.03 (-0.56–0.46)	0.05 (-1.13–0.44)	>0.9
**No preserving agents**	0.20 (-0.59–1.15)	-0.01 (-1.62–0.69)	0.12

Five outliers, labeled A to E in Fig 4, are the results of high CT values at which PCR quantification becomes inaccurate.

## Discussion

In this study, we aimed to determine the most optimal and practical conditions to handle and store urine samples for the purpose of molecular analysis with specific focus on the analysis of hmDNA. Our results demonstrate that addition of EDTA to urine samples collected from cancer patients significantly reduced DNA degradation. EDTA reduced DNA degradation at room temperature during a period of at least 7 days (*p*<0.001). On the contrary to the findings at RT, the use of preserving agents did not result in a statistically significant difference in DNA degradation after storage at 4°C (*p* = 0.18). This suggests that the addition of preserving agents might be omitted when samples are stored at 4°C. Yet, in this study urinary samples were immediately stored at 4°C after collection. In case of collection at patient’s homes, there is no control over the time period that a sample is left at RT prior to, or after, storage at 4°C (e.g. transport to the laboratory). Therefore, addition of EDTA might still be considered for ambulant collection and storage at 4°C.

The effect of EDTA on the degradation of conventional urinary DNA (i.e. without preceding bisulfite conversion for methylation analysis as studied herein) has been demonstrated in two other studies [15, 16]. Cannas *et al*. measured urinary DNA after 28 days of storage at 4°C with and without the addition of EDTA. Addition of EDTA to urine samples stored at 4°C resulted in an average loss of DNA of 1.6%, whereas storage at 4°C without the addition of EDTA resulted in a 45% loss of DNA [15]. Although in the present study we did not find a difference in the loss of DNA in case EDTA was added to samples stored at 4°C, this is most likely explained by the shorter period of storage in our study (28 days versus 7 days).

Milde *et al*. also investigated the effect of EDTA on 10 urine samples stored at -20°C and at RT and found that the inhibiting effect of EDTA on DNA degradation was superior to the inhibiting effect of cooling (-20°C). Moreover, the addition of EDTA resulted in less DNA degradation in samples stored at -20°C for 72 days as compared to samples stored without a preserving agent [16].

To determine whether EDTA should be added to urine samples prior to hmDNA analysis, it is important to investigate whether degradation of hmDNA (*RASSF1A*) follows the same pattern as the degradation of bisulfite modified human DNA in general (*ACTB*). We found that methylated *RASSF1A* indeed followed similar patterns of DNA degradation as *ACTB*. There were some outliers (Fig 4) with high CT values at which PCR reproducibility is distorted. These data points represented samples without preserving agents and samples to which only PenStrep was added. Since no outliers were observed in any of the aliquots to which EDTA was added, we recommend to add EDTA for hmDNA analysis in urine. By addition of EDTA urine samples can be stored at RT or 4°C for up to 7 days prior to DNA isolation, allowing for reliable hmDNA analysis. In our study the addition of antibiotics (PenStrep) did not preserve DNA at RT or 4°C. The rationale of adding antibiotics was to decrease bacterial growth and thereby reduce DNA degradation. It should be mentioned that in this study all samples were collected in the hospital, whereas upon collection at a patient’s home bacterial contamination seems more likely. As the addition of PenStrep did not negatively influence PCR results, we recommend to add antibiotics in case of ambulant collection.

To our knowledge, this is the first study to assess the preservation of DNA in urine for the purpose of hmDNA detection, which requires bisulfite modification prior to DNA amplification, a process associated with extensive degradation of DNA [6]. Another novelty of our study is the inclusion of oncologic patient samples representing clinical practice, whereas most studies use specimens collected from healthy individuals only. A limitation of our study includes the storage period of 7 days. Accordingly, no recommendations for long-term storage can be provided. On the other hand, in our experience, 7 days is usually suffice to allow for transportation of the sample to a laboratory. Furthermore, we did not investigate the degradation of other components in urine such as RNA or proteins. Whether EDTA also acts as a preservative for purposes other than methylation analysis, can therefore not be answered from our data. Additionally, although urine aliquots of individual patients were processed simultaneously for DNA isolation and bisulfite conversion, we cannot fully exclude a confounding effect of variations in DNA degradation upon bisulfite conversion. However, DNA concentration measurements of non-bisulfite treated DNA also demonstrated a preserving effect of EDTA. This suggests that EDTA has similar effects on DNA regardless of the methylation status or bisulfite modification. Finally, for the pilot study we used three samples only. Therefore, our further testing was based on visual inspection of the graph (Fig 2), instead of a statistical analysis.

The finding that EDTA can preserve DNA and improves the reliability of hmDNA analysis on urine samples, paves the way for molecular urine diagnostics studies using ambulant sample collection in the field of oncology. In future studies, it would be interesting to determine the optimal volume of urine to provide sufficient DNA for the analysis of interest. Furthermore, optimal storage conditions for a longer period of time (e.g. for the purpose of a biobank) need further investigations.

## Conclusions

Addition of EDTA offers an inexpensive preserving agent for temporary urine storage at RT or 4°C allowing for reliable hmDNA analysis. Although at 4°C DNA is also retained without the use of preserving agents, addition of EDTA improved the reliability of PCR results. Penicillin Streptomycin can safely be added in case bacterial overgrowth is expected without affecting DNA yield.

## Supporting information

S1 File CT_*ACTB*_ per sample at various conditions. (DOCX)

S2 File CT values per sample at various conditions. (DOCX)

S1 Table CT difference of *RASSF1A* and *ACTB* at day 7 compared to day 0, in samples of NSCLC patients versus bladder cancer patients at various conditions. (DOCX)

S2 Table DNA concentrations measured by Qubit^™^ dsDNA HS Assay (Invitrogen) in a subset of clinical samples comparing day 0 and day 7, with or without addition of EDTA. (DOCX)
