# Supplementary material for: A protocol for urine collection and storage prior to DNA methylation analysis
Source: PLoS One. 2018 Aug 24;13(8):e0200906. doi: 10.1371/journal.pone.0200906 (PMC6108475; doi:10.1371/journal.pone.0200906)
Supplement: S1 Table — (DOCX) [file pone.0200906.s003.docx]

| Condition at RT | ∆CT*_ACTB_*  _median_  _(IQR)_ | | *p*-value  (Mann Whitney *U* test) | ∆CT*_RASSF1A_*  _median_  _(IQR)_ | | *p*-value  (Mann  Whitney *U* test) |
| --- | --- | --- | --- | --- | --- | --- |
|  | BC | NSCLC |  | BC | NSCLC |  |
| EDTA | 0.57  (-0.22-1.05) | -0.24  (-1.22-0.71) | 0.2 | 0.16  (-0.66-0.69) | 0.16  (-0.85-0.83) | >0.9 |
| PenStrep | 3.33  (1.73-4.66) | 3.27  (2.25-4.69) | 0.9 | 2.71  (1.34-5.07) | 3.31  (0.35-3.93) | 0.8 |
| EDTA + PenStrep | 0.64  (-0.32-1.03) | -0.10  (-1.28-1.19) | 0.6 | 0.23  (-0.52-0.75) | 0.19  (-0.80-1.67) | 0.8 |
| No preserving agents | 4.28  (2.42-6.24) | 3.46  (2.21-4.70) | 0.4 | 3.33  (-0.22-4.16) | 3.19  (0.23-7.91) | 0.7 |
| Condition at 4°C |  | |  |  | |  |
| EDTA | 0.55  (-0.28-0.73) | 0.44  (-0.63-4.11) | >0.9 | 0.15  (-0.64-0.52) | 0.48  (-0.38-5.06) | 0.2 |
| PenStrep | 0.26  (-0.25-0.87) | 0.71  (-1.11-2.87) | 0.4 | 0.12  (-0.23-1.19) | 0.61  (-0.74-10.8) | 0.4 |
| EDTA + PenStrep | 0.08  (-0.92-0.85) | -0.03  (-1.37-0.49) | 0.7 | 0.01  (-0.67-0.33) | -0.19  (-1.04-0.74) | >0.9 |
| No preserving agents | -0.10  (-1.34-0.76) | 0.28  (-1.34-0.76) | 0.6 | 0.07  (-0.67-1.08) | 0.50  (-0.99-1.25) | 0.5 |
