# Supplementary material for: A protocol for urine collection and storage prior to DNA methylation analysis
Source: PLoS One. 2018 Aug 24;13(8):e0200906. doi: 10.1371/journal.pone.0200906 (PMC6108475; doi:10.1371/journal.pone.0200906)
Supplement: S2 Table — (DOCX) [file pone.0200906.s004.docx]

|  | **Sample at day 0** | **Concentration day 7 (w/o EDTA)** | **Concentration day 7 (EDTA added)** |
| --- | --- | --- | --- |
|  | **DNA (ng/ul)** | **DNA (ng/ul)** | **DNA (ng/ul)** |
| **NSCLC 1** | 6,8 | 0,746 | 5,62 |
| **NSCLC 2** | 61,4 | 2,74 | 77,2 |
| **NSCLC 3** | 3,86 | 0,776 | 3,86 |
| **NSCLC 4** | 40,6 | 3,2 | 34,2 |
| **NSCLC 6** | 6,14 | 1,58 | 9,25 |
|  |  |  |  |
| **BC 1** | 6,4 | 6,76 | 6,44 |
| **BC 2** | 2,18 | 0,544 | 2,82 |
| **BC 5** | 3,84 | 0,618 | 5,52 |
| **BC 7** | 24,7 | 2,36 | 10,4 |
