# Supplementary material for: A protocol for urine collection and storage prior to DNA methylation analysis
Source: PLoS One. 2018 Aug 24;13(8):e0200906. doi: 10.1371/journal.pone.0200906 (PMC6108475; doi:10.1371/journal.pone.0200906)
Supplement: Supplementary file 3 [file pone.0200906.s001.docx]

Room temperature

	No additives				EDTA				Commercial Buffer		
	volunteer				volunteer				volunteer		
day	A	B	C	day	A	B	C	day	A	B	C
0	29,32	25,41	29,97	0	29,32	25,41	29,97	0	29,32	25,41	29,97
1		26,96	31,23	1		25,45	29,50	1		25,42	28,96
2	33,28	27,99	31,75	2	29,74	25,79	29,99	2	28,35	25,67	29,12
7	36,92	29,61	33,96	7	30,99	26,67	30,02	7	29,29	28,63	28,94
28		33,08	34,67	28		28,57	30,58	28		25,53	29,10

4°c

	No additives				EDTA				Commercial Buffer		
	volunteer				volunteer				volunteer		
day	A	B	C	day	A	B	C	day	A	B	C
0	29,31955147	25,40600777	29,97045517	0	29,31955	25,40601	29,97046	0	29,31955	25,40601	29,97046
1		26,04500008	30,26187134	1		25,47124	30,62203	1		26,21331	30,05748
2	29,84227943	26,20745277	30,21648407	2	29,77497	30,44277	25,73433	2	30,55886	25,59614	29,99116
7	31,93845749	27,66251945	31,29976463	7	29,47902	26,41558	30,01547	7	29,45304	29,18384	28,80257
28		28,45466232	32,95298386	28		25,67677	29,30158	28		25,5526	28,75455

minus 20 °C

	No additives				EDTA				Commercial Buffer		
	volunteer				volunteer				volunteer		
day	A	B	C	day	A	B	C	day	A	B	C
0	29,31955147	25,40600777	29,97045517	0	29,31955	25,40601	29,97046	0	29,31955	25,40601	29,97046
1		27,08790207	32,08187866	1		25,9175	29,90369	1	30,4559	25,77854	29,83691
2	30,56814003	26,68099022	30,92947578	2	29,17663	26,13689	30,37105	2	29,33302	25,43984	29,86278
7	29,51961899	27,13237381	31,09384155	7	30,03946	26,24377	30,25853	7	30,38991	28,60377	30,39649
28		25,51181412	30,66848183	28		24,59406	30,05734	28		25,66706	30,03612

minus 80 °C

	No additives				EDTA				Commercial Buffer		
	volunteer				volunteer				volunteer		
day	A	B	C	day	A	B	C	day	A	B	C
0	29,31955147	25,40600777	29,97045517	0	29,31955	25,40601	29,97046	0	29,31955	25,40601	29,97046
1		26,99906731	30,78939629	1		26,20821	30,42424	1	29,85868	25,88102	29,93336
2	31,85763168	25,93401146	30,32304764	2	28,99316	26,4865	30,30493	2	28,93564	25,67177	29,84802
7	29,46466446	27,49962425	31,03984451	7	30,69544		29,90556	7	29,87646	30,08153	29,9211
28		26,49980545	29,96236229	28			30,00845	28		25,24282	29,99104
