# Supplementary material for: A protocol for urine collection and storage prior to DNA methylation analysis
Source: PLoS One. 2018 Aug 24;13(8):e0200906. doi: 10.1371/journal.pone.0200906 (PMC6108475; doi:10.1371/journal.pone.0200906)
Supplement: Supplementary file 4 [file pone.0200906.s002.docx]

| | | | CT Values per sample per condition | | | | | | |
|---|---|---|---|---|---|---|---|---|---|
| | **day0** | **edtaRT** | **EDTA4C** | **PSRT** | **PS4C** | **EPSRT** | **EPS4C** | **BRT** | **B4C** |
| NSCLC1 A | 24,855 | 25,405 | 25,900 | 31,381 | 25,752 | 25,924 | 25,644 | 30,188 | 25,848 |
| NSCLC1 F | 30,76127 | 31,288 | 31,637 | 33,793 | 30,971 | 30,870 | 31,322 | 35,112 | 31,138 |
| NSCLC2 A | 27,49617 | 21,107 | 22,182 | 27,751 | 21,067 | 20,966 | 21,345 | 28,035 | 21,715 |
| NSCLC2 F | 45 | 30,182 | 31,317 | 34,974 | 30,244 | 30,367 | 30,341 | 35,109 | 30,355 |
| NSCLC3 A | 28,57656 | 26,751 | 45 | 29,916 | 26,846 | 26,297 | 26,491 | 30,148 | 26,602 |
| NSCLC3 F | 31,6508 | 30,495 | 45 | 31,810 | 29,940 | 29,993 | 30,137 | 31,980 | 30,057 |
| NSCLC4 A | 25,37582 | 25,195 | 25,417 | 29,452 | 25,839 | 25,147 | 25,277 | 29,551 | 25,943 |
| NSCLC4 F | 29,961 | 30,126 | 30,408 | 34,259 | 30,568 | 30,149 | 30,012 | 33,313 | 30,659 |
| NSCLC5 A | 25,29632 | 25,010 | 31,274 | 27,848 | 32,400 | 24,834 | 24,587 | 27,719 | 25,284 |
| NSCLC5 F | 31,310 | 31,228 | 36,833 | 34,867 | 39,275 | 31,837 | 31,102 | 33,960 | 32,460 |
| NSCLC6 A | 24,67763 | 24,896 | 24,970 | 28,498 | 27,438 | 26,222 | 25,831 | 29,171 | 25,366 |
| NSCLC6 F | 31,404 | 31,655 | 31,536 | 33,854 | 45 | 32,642 | 32,423 | 39,704 | 31,902 |
| NSCLC7 A | 25,47655 | 24,896 | 24,970 | 28,498 | 27,438 | 26,222 | 25,831 | 29,171 | 25,366 |
| NSCLC7 F | 30,544 | 31,655 | 31,536 | 33,854 | 45 | 32,642 | 32,423 | 39,704 | 31,902 |
| NSCLC8 A | 25,95253 | 29,473 | 29,442 | 32,690 | 29,155 | 30,267 | 26,348 | 32,441 | 29,899 |
| NSCLC8 F | 30,678 | 38,287 | 35,272 | 36,765 | 34,842 | 36,653 | 31,135 | 38,200 | 35,790 |
| NSCLC9 A | 27,12298 | 26,098 | 26,165 | 29,852 | 26,218 | 26,175 | 25,989 | 30,193 | 25,992 |
| NSCLC9 F | 32,877 | 31,174 | 31,524 | 33,425 | 31,597 | 31,393 | 31,158 | 33,003 | 30,968 |
| NSCLC10 | 23,9548 | 25,157 | 24,532 | 27,486 | 24,488 | 23,987 | 24,005 | 27,184 | 24,532 |
| NSCLC10 | 28,842 | 29,396 | 29,326 | 32,220 | 29,006 | 28,959 | 28,657 | 32,032 | 29,326 |

***abbreviations***

| | |
|---|---|
| BC | bladder cancer |
| RT | room temperature |
| 4c | 4 degrees |
| EPS | EDTA and PenStrep |
| B | no additives |

| | day0 | edtaRT | EDTA4C | PSRT | PS4C | EPSRT | EPS4C | BRT | B4C |
|---|---|---|---|---|---|---|---|---|---|
| | | | **CT Values per sample per condition** | | | | | | |
| BC 1 actb | 24,356 | 24,924 | 25,778 | 27,69 | 25,415 | 23,922 | 26,147 | 27,836 | 25,581 |
| BC 1 rassf1a | 29,525 | 30,038 | 30,179 | 34,547 | 30,664 | 29,798 | 30,488 | 34,748 | 30,755 |
| BC 2 actb | 26,159 | 27,061 | 26,875 | 35,511 | 26,741 | 27,622 | 27,421 | 30,687 | 26,855 |
| BC 2rassf1a | 30,279 | 30,922 | 30,75 | 35,496 | 31,608 | 32,107 | 30,864 | 34,193 | 30,481 |
| BC 3actb | 26,827 | 26,265 | 27,38 | 30,232 | 26,477 | 27,467 | 26,907 | 35,369 | 26,213 |
| BC3 rassf1a | 30,797 | 30,407 | 30,168 | 35,613 | 30,986 | 31,523 | 29,624 | 34,443 | 30,277 |
| BC4actb | 22,462 | 21,727 | 22,897 | 23,656 | 21,344 | 21,199 | 22,905 | 23,412 | 26,118 |
| BC4rassf1a | 27,375 | 26,942 | 27,583 | 27,604 | 27,3 | 27,545 | 27,346 | 27,103 | 28,996 |
| BC5actb | 27,833 | 24,521 | 28,383 | 28,737 | 25,899 | 24,907 | 22,81 | 29,199 | 25,372 |
| BC5rassf1a | 30,412 | 29,062 | 33,475 | 32,122 | 29,754 | 29,516 | 30,033 | 34,312 | 29,5 |
| BC7actb | 21,839 | 23,347 | 22,532 | 24,572 | 22,517 | 21,946 | 22,281 | 28,57 | 21,854 |
| BC7rassf1a | 31,446 | 34,24 | 31,072 | | 32,777 | 31,996 | 31,488 | | 32,477 |
| BC9 actb | 22,866 | 23,365 | 23,601 | 27,41 | 23,078 | 23,651 | 21,48 | 28,621 | 23,361 |
| BC 9 rassf1a | 31,302 | 31,541 | 31,492 | 33,653 | 31,351 | 31,481 | 31,44 | 36,182 | 30,709 |
| BC 11 actb | 21,567 | 21,684 | 21,144 | 24,118 | 21,418 | 22,365 | 21,659 | 25,274 | 21,466 |
| BC 11 rassf1a | 21,717 | 21,799 | 21,825 | 23,838 | 21,634 | 22,52 | 21,959 | 24,727 | 21,74 |
| BC 8 actb | 45 | 24,726 | 25,454 | 30,105 | 23,626 | 24,462 | 24,727 | 33,477 | 24,280 |
| BC 8 rassf1a | 32,406 | 26,532 | 26,915 | 29,069 | 25,841 | 26,074 | 25,969 | 32,208 | 26,596 |
| BC 12 actb | 27,29423 | 28,495 | 27,165 | 32,079 | 27,553 | 27,098 | 27,022 | 31,579 | 25,669 |
| BC 12 rassf1a | 30,610 | 31,461 | 29,962 | 33,683 | 30,803 | 30,221 | 30,110 | 33,323 | 30,729 |

***abbreviations***

| | |
|---|---|
| BC | bladder cancer |
| RT | room temperature |
| 4c | 4 degrees |
| EPS | EDTA and PenStrep |
| B | no additives |
